# The Week's Work

**Published:** 1909-12-18

**Authors:** 


### The Week's Work.

### THE LEICESTER INFIRMARY.

In Sir Henry Burdett's report on this hospital, which was published in our issue last week, it was stated that he failed to find the cost of each in- and out-patient for 1908 published in the report. We find that this cost is set out on pages 30 and 31 of the report for 1908 in a series of tables dealing with the financial statistics of the year. These tables are full and exhaustive, and we could wish that the form of them might be adopted by hospitals generally. The report contains a classified list of contributions other than those to the Extension Fund received by the infirmary from county districts, together with the cost of the treatment of the patients admitted during the year from each such district. It would be interesting to have the totals of contributions and of cost for the whole of these districts added to the tables in future reports. We believe that if the facts embodied in the tables could be brought to the personal notice of every resident in each district, the revenue from voluntary contributions must materially increase. We are glad to learn, too, that the report will contain photographs of the interior of the Infirmary, to which will be added a complete plan of the buildings when the reconstruction scheme is completed. We have already expressed our appreciation of the efficiency of the administration of this important county hospital, where the true hospital spirit prevails, and where every worker is whole-heartedly devoted to the best interests of the patients and the promotion of the reputation of the hospital.

### THE MIDHURST SANATORIUM.

In our issue of the 13th ult. we called attention to an article in *John Bull* on The King Edward VII Sanatorium at Midhurst. Having shown that the main indictment drawn up by "An Ex-Patient" was frivolous and unimportant, we directed attention to three points which demanded inquiry. We have now had an opportunity of inspecting this Sanatorium, and can deal with these points completely. The first relates to the closing of the writing-room until an hour before post time. We find that this statement loses its point from the circumstance that there are later posts than that mentioned, and that the patients are allowed to write letters with a pencil or a stylographic pen in their own rooms. Indeed, we found the facilities afforded for writing letters to be ample and satisfactory. With reference to ringworm, which has affected some of the patients, the most drastic measures have been taken, and have proved effective. At the time of our visit there were no cases of ringworm in the Sanatorium. The epidemic was thoroughly grappled with, expert advice was sought, and there is every reason to hope that nothing of the kind will occur in future, and that this troublesome complaint, which is sometimes met with in sanatoria, has been eradicated. Lastly, the charge of dampness is exaggerated, if it is not altogether without foundation. When we inspected the Sanatorium it was a very wet day, and we found the patients sitting in their rooms with the windows open, which permitted the rain to moisten a portion of the floor. The pillows and linen were, however, perfectly dry, and so were the walls of the rooms. We questioned the patients, who all of them assured us that they had never had any cause to complain of the dampness of the pillows or bedding, and several of them had been inmates for a considerable time. Our inspection gave us the impression that the patients were well cared for, happy, and comfortable. The Sanatorium is well found: the living-rooms and dormitories were in good order, and the quality of the food was good, too. Had *John Bull* attacked the architectural features of the building and its cost, our contemporary would have had abundant justification. After a careful inspection, it is but fair to the authorities to state, however, that the indictment drawn up by "An Ex-Patient" will not bear examination. We found the charges to be either frivolous or without adequate foundation. This Sanatorium is doing good work, and everybody connected with the administration is evidently desirous to promote the comfort, recovery, and welfare of the patients to the fullest possible extent.

### A MEDICAL STAFF DISREGARDED.

An unfortunate situation has arisen at the Cancer Hospital, Fulham Road, in connection with an appointment to the surgical staff. A vacancy for assistant surgeon recently occurred in consequence of the promotion of the senior assistant surgeon to a vacancy on the senior staff. The appointment was duly advertised, and from the applicants the medical committee selected a gentleman who has for three years past filled the post of Registrar. The recommendation of this surgeon to the House Committee is understood to have been unanimous; but the latter saw fit to override it by appointing one of the other candidates. The Medical Committee has promptly addressed a letter of protest to the House Committee, and there, for the moment, the matter stands. There is, however, an aspect of the dispute which is wider than the domestic circle of the management of the Cancer Hospital. That is, to what extent and in what circumstances a Governing Board or Committee is justified in thus picking a quarrel with its medical staff. In the instance under review there is nothing that can possibly be urged against the medical staff's selection, for during three years of office they have naturally had the best of opportunities of studying the Registrar's ability and capacity, which are indisputable. Without, therefore, any expert knowledge of the qualifications of the various candidates, the lay committee has ventured to force upon the medical staff a stranger as a colleague in a manner which is open to the most adverse criticism and to suspicion of favouritism or influence. A similar course, curiously enough, was also taken by the House Committee at the time of the last election but one to the surgical staff, and was allowed to pass without any effective protest from the Medical Committee, which is paying for its then weakness by a repetition of the insult. There is a further consideration which may possibly have escaped the House Committee, and this is that it may not prove easy to attract suitable candidates in future for the post of registrar when the way in which the present holder of the post has been treated becomes generally known. It would, of course, be foolish to regard registrars as in any way entitled to vacancies upon the staff; and there is, in fact, no danger whatever of any such custom becoming established. But when a registrar who has served his hospital well is a candidate for election, his claims should not be disregarded unless there is some other candidate of definitely superior ability and experience: least of all should the Medical Committee's recommendation be treated in this contemptuous way. The ability to discriminate between the qualifications, academic and practical, of medical men is not possessed by one layman in a hundred; and the Governors' proper course, if they were dissatisfied with the nomination of the staff, was to refer the matter back to the latter for reconsideration or explanation.

### A COMMON-SENSE REPORT.

The example set by the management of the Royal Hospital for Incurables, Putney, which has just issued a Christmas appeal booklet, is one that might with advantage be followed by general and special hospitals. Ordinary annual reports are in the vast majority of cases very dry reading. No attempt whatever is made to interest subscribers and others in the work that is being accomplished by presenting the account of such work in a readable and attractive manner. The result is that those who receive the annual reports hardly ever read them, contenting themselves with a glance at the financial addenda or the special appeals. That is a matter which urgently calls for consideration on the part of every hospital secretary and superintendent. The Putney Hospital, in its little booklet "How the Money Goes," has attempted to present its facts in an easily digestible and attractive manner. The account of the work done is clear, concise, and written in an admirably graphic manner. The reader is interested from the start, and that interest is sustained right to the very end of the report, which, it must be added, is exceedingly well got up, so that it is a pleasure to read and study it. We congratulate the management on its enterprise, and trust that the innovation will be a great success, and win the hospital many new friends and subscribers.

### THE VALUE OF ILLUSTRATION.

We have always felt that what is needed in a hospital report is illustration. Not necessarily pictorial illustration but illustration of a kind that the average individual who is utterly unacquainted with hospital work and methods can readily understand and correctly appreciate. The bare mention of the fact that the average daily cost per patient has declined so many pence during the past year is a detail which such a lay reader cannot grasp. It leaves him cool, for he has no idea whatever, especially if he be a person who is not quick at figures, of its meaning to the finances of the institution. Baldly presented to him in so many figures, it is unattractive and useless as a forcible appeal. How much more powerful, however, would such facts be if they were illustrated by analogy to daily household expenses? It would not be a difficult task to compile a table showing the average daily cost per head in a working man's family, a middle-class family, and so on, and compare and contrast these figures with the hospital statistics. these last, we feel convinced, will be rendered much more intelligible presented in this fashion. The same may be said of the gross returns issued by the dispensary, out-patient and casualty department, and so on. What lay reader can comprehend what is meant by an annual consumption of so many hundreds of pounds of potassium iodide or so many tons of malt extract? The enormous expense to the hospital, however, is easily conceivable, even by the lay mind, when these statistics are amplified by reference to their concrete mass. So many pounds of pot. iod. means so much iodine, so many tons of malt extract means so much wheat, and fills so many casks of such and such dimensions; the bandages of the hospital laid end to end will go so far or form a road so wide! These may seem ridiculous comparisons worthy only of the penny weeklies, but let us not forget that a large share of support must come from the readers of the penny weeklies, and it is good policy to interest them. The hospital report of the future, if the voluntary system is to endure, will have to be different from the present-day stereotyped statistical summary. It will have to be alive, "with guts in it," as the journalese term is, and hospital boards might do worse than secure the services of a good literary man to edit their reports for them and present their facts in a more convincing and less bald fashion than is nowadays customary.

### THE DRUG MARKET.

*A propos* of the price of spirit, the wholesale drug houses have intimated that they will allow a rebate on all spirituous preparations purchased from them from now until the date of the passing of a Finance Bill for the year ending March 31, 1910, corresponding to the amount allowed to them by the Customs and Excise authorities. That is to say, tinctures and the like preparations will be invoiced at the prices at present quoted, but should the extra duty of 3s. 9d. per proof gallon not be sanctioned by the Finance Act, an amount will be deducted corresponding to the extra charge which has been made. As was expected, glycerine refiners have advanced their prices, and there seems every probability that before long prices will be still higher. The advance is mainly due to the increased demand for crude glycerine for the manufacture of explosives. A definite price is now quoted for santonin, but from the apearance of the market it seems likely that the value will advance. In the note which appeared under this heading last week, "gum ammoniacum" was printed by mistake for "gum asafœtida." The drug market is now becoming quieter each week, as buyers do not care to take in a supply of drugs larger than is necessary to satisfy their requirements until the end of the year.

